# Analysis of the microbial species, antimicrobial sensitivity and drug resistance in 2652 patients of nursing hospital

**DOI:** 10.1016/j.heliyon.2020.e03965

**Published:** 2020-05-18

**Authors:** Wu Yang, Xu Ji

**Affiliations:** a Administration Department of Shanghai AIYIDE Gaoping Nursing hospital, No.847 gaoping road, Jing an distinct, Shanghai, 200043, China; b Ophthalmology Department of Shanghai East hospital, Tongji University School of Medicine, No.1800 yuntai Road, Pudong distinct, Shanghai, 200123, China

**Keywords:** Microbial species, Infectious disease, Antimicrobial, Nursing, Drug resistance, Nursing hospital, Antimicrobial sensitivity

## Abstract

**Objective:**

To understand the microbial species, antimicrobial sensitivity and drug resistance of patients in the nursing hospital, to bring clinical guiding significance to the use of antimicrobial in the nursing hospital.

**Methods:**

A retrospective analysis of bacterial and drug sensitivity report of 2652 patients in nursing hospitals.

**Results:**

There were 2652 cases of bacterial and drug sensitivity results, including 1202 positive cases, 652 females accounted for 54.24%, and 550 males accounted for 45.76%, concentrated in 70–99 years old. There were 57 kinds of bacteria, 303 cases of Gram-positive bacteria accounted for 25.21%, 808 cases of Gram-negative bacteria accounted for 67.22%, and fungi accounted for 7.57%. The positive samples were mainly from urine (35.94%) and sputum (48.59%). The main Gram-positive bacteria in the positive samples were Staphylococcus aureus (53.14%), Enterococcus faecalis (12.87%), Staphylococcus epidermidis (10.23%), Staphylococcus haemolyticus (6.93%), Enterococcus faecium (3.96%). The main Gram-negative bacteria in the positive samples were E. coli (35.27%), Pseudomonas aeruginosa (20.67%), Klebsiella pneumoniae (13.99%), Proteus mirabilis (8.66%), Bowman The main fungi in the positive samples of the bacillus (5.57%) were Candida albicans (59.34%), Candida albicans (16.48%), and Candida glabrata (14.29%). Among the Gram-positive bacteria, Enterococcus faecium and Enterococcus faecalis are easily detected in urine. Staphylococcus haemolyticus and Staphylococcus epidermidis are easily detected in sputum. Staphylococcus aureus is easily detected in sputum and secretions. Among the Gram-negative bacteria, E. coli and Klebsiella pneumoniae are easily detected in sputum and urine. Proteus mirabilis is easily detected in the urine. Pseudomonas aeruginosa and Acinetobacter baumannii are easily detected in sputum. Among the fungi, Candida albicans, Candida bulgaricus, and Candida glabrata are easily detected in sputum. There is a significant statistically difference in the distribution of P. aeruginosa in all age groups. The resistance rate of Staphylococcus aureus to levofloxacin and penicillin are above 90%, the resistance rate to clindamycin and erythromycin are above 80%, the resistance rate to gentamicin is above 70%, and vancomycin and linezolid The sensitivity rate of amine is 100.0%, the sensitivity rate to rifampicin and teicoplanin are above 90%, and the sensitivity to compound sulfamethoxazole and streptomycin are above 80%. Staphylococcus aureus resistant to vancomycin and linezolid are not found this time. The resistance rate of E. coli to compound sulfamethoxazole is above 60%, the resistance rate to cefuroxime, ceftriaxone, levofloxacin, and ciprofloxacin are above 70%, and the resistance rate to cefazolin is above 80%. The resistance rate to ampicillin is above 90%, the sensitivity rate to nitrofurantoin, minocycline and cefoxitin are above 60%, and the sensitivity to cefoperazone sulbactam and piperacillin tazobactam is above 70%. The sensitivity rate of ropamine, imipenem and amikacin are above 80%. The resistance rate of Proteus mirabilis to nitrofurantoin is 100%, the resistance rate to ampicillin are above 90%, and the resistance rate to compound sulfamethoxazole, levofloxacin and ciprofloxacin are above 80%. For cefazolin and gentamicin The rate of resistance is above 70%. The sensitivity rate to cefoxitin, imipenem and amikacin are above 80%, and the sensitivity to meropenem, cefoperazone sulbactam, and piperacillin tazobactam are above 90%. Klebsiella pneumoniae to cefazolin, gentamicin, ampicillin, cefuroxime, ceftazidime, cefoxitin, ceftriaxone, ampicillin sulbactam, compound sulfamethoxazole, levofloxacin, ciprofloxacin The drug resistance rate is above 60%, the sensitivity rate to amikacin is only above 60%, and the sensitivity rate to imipenem and meropenem is only above 50%. Pseudomonas aeruginosa tested resistance to antimicrobial is below 50%, sensitivity to ceftazidime, cefepime, meropenem, imipenem, amikacin are above 60%, sensitivity to polymyxin B is above 90%, the sensitivity rate of tigecycline is 100%. The resistance rate of Candida glabrata to fluorocytosine is above 60%, the sensitivity rate to amphotericin B, fluconazole and itraconazole are above 60%, and the sensitivity rate of voriconazole is above 90%.

**Conclusion:**

There is a difference between this data and the bacteria data in Shanghai. It is necessary to understand the bacteria sensitivity rate and resistance rate of antimicrobial in the nursing hospital, and it has certain guiding significance for the clinical of the nursing hospital.

## Teaser

Bacteria; Infectious disease; Antimicrobial; Nursing; Drug resistance; Nursing hospital; Antimicrobial sensitivity.

## 1 Introduction

According to the basic standards of nursing hospital (2011 edition) published by the Ministry of Health,PRC, the Nursing hospital provides medical care and rehabilitation for long-term hospitalized patients, patients with advanced palliative care, patients with chronically ill, elderly people who cannot take care of themselves or their families, and other patients who need long-term medical care services.

The tasks undertaken by the nursing hospital are different from those General hospitals. nursing hospitals are relatively insufficient in medical human resources, equipment, antimicrobial Drugs. At the same time, most of the patients in the nursing hospital have several chronic diseases, long duration of hospital stay, old age, and most have received the antimicrobial treatment in General hospitals. Therefore, mastering microbial species of hospitalized patients in the nursing hospital and the sensitivity and resistance of antimicrobial drugs in clinical can guide the usage of the antimicrobial drug in patients with early-onset.

The objective of this article is to understand the microbial species microbial species, antimicrobial drugs sensitivity and resistance from 2652 cases of patients in the nursing hospital, to bring a clear view of nursing hospital doctors.

## 2 Information and methods

### 2.1 Research materials

A retrospective analysis of 2,652 cases of hospitalized patients in five nursing hospitals of Shanghai from January to June 2019. Among them, there were 1202 effective results, 1450 cases of ineffective results, 987 cases have no microbial been found, 379 cases of non-pathogenic bacteria and 84 cases have been polluted. This article analyzes and discusses ineffective results.

### 2.2 Instruments and reagents

DL-96A bacteria identification and drug sensitivity analyzer produced by Zhuhai Dill Bioengineering Co., Ltd., DL-Bt240 automatic blood culture device.

### 2.3 Methods

Cultivate and drug sensitivity was carried out by the National Clinical Laboratory Procedures. The drug susceptibility test was carried out by the paper diffusion methods recommended by the American Committee for Clinical Laboratory Standards (CLSI), and the results were judged according to the CLSI-M100 standard.

### 2.4 Statistical processing

Using SPSS 19.0 statistical software, the count data were expressed as frequency (n), and the chi-square test was used for comparison between groups. P < 0.05 was considered statistically significant.

## 3 Results

### 3.1 General information

There were 2652 cases results, of which 1202 were positive.

3.1.1 The gender distribution of a total of 652 female cases, accounted for 54.24%. 550 males cases, accounted for 45.76%.
3.1.2 The age distribution of total ranged from 40 to 110 years old, the youngest was 49 years old, and the eldest was 105 years old. The average age was 82.77 ± 9.09 years old, concentrated in 70–99 years old. Among them, 70–79 years old accounted for 18.55%, 80–89 years old accounted for 48.09%, and 90–99 years old accounted for 21.21% (Table 1).

**Table 1:** Total age distribution.

Age range	Frequency	Constituent ratio
100~110	20	1.66%
90~99	255	21.21%
80~89	578	48.09%
70~79	223	18.55%
60~69	110	9.15%
50~59	14	1.16%
40~49	2	0.17%
total	1202	100.00%

### 3.2 Distribution

3.2.1 A total of 57 microbial species were detected in all positive samples, of which 303 cases were Gram-positive microbial species accounted for 25.21%, 808 cases of Gram-negative microbial species accounted for 67.22%, and fungi accounted for 7.57%.
3.2.2 Positive samples were mainly from urine (35.94%) and sputum (48.59%).
3.2.3 The main Gram-positive bacteria in the positive samples were Staphylococcus aureus (53.14%), Enterococcus faecalis (12.87%), Staphylococcus epidermidis (10.23%), Staphylococcus haemolyticus (6.93%), Enterococcus faecium (3.96%).
3.2.4 The main Gram-negative bacteria in the positive samples were E. coli (35.27%), Pseudomonas aeruginosa (20.67%), Klebsiella pneumoniae (13.99%), Proteus mirabilis (8.66%), Bowman Bacillus (5.57%)
3.2.5 The main fungi in the positive samples were Candida albicans (59.34%), Candida bulgaricus (16.48%), Candida glabrata (14.29%).
3.2.6 Among the main Gram-positive bacteria, Enterococcus faecium and Enterococcus faecalis are easily detected in urine. Staphylococcus haemolyticus and Staphylococcus epidermidis are easily detected in sputum. Staphylococcus aureus is easily detected in sputum and secretions (Table 2).

**Table 2:** Distribution of specimens of major Gram-positive bacteria.

Bacterial species	Bacterial name	type of sample	Constituent ratio
G+	Enterococcus faecium	urine	75.00%
G+	Enterococcus faecium	blood-Aerobic	16.67%
G+	Enterococcus faecium	sputum	8.33%
G+	Staphylococcus haemolyticus	sputum	47.62%
G+	Staphylococcus haemolyticus	blood- Anaerobic	19.05%
G+	Staphylococcus haemolyticus	blood-Aerobic	14.29%
G+	Staphylococcus haemolyticus	Secretion	9.52%
G+	Staphylococcus haemolyticus	urine	9.52%
G+	Enterococcus faecalis	urine	79.49%
G+	Enterococcus faecalis	sputum	15.38%
G+	Enterococcus faecalis	Secretion	5.13%
G+	Staphylococcus epidermidis	sputum	70.97%
G+	Staphylococcus epidermidis	blood-Aerobic	12.90%
G+	Staphylococcus epidermidis	Secretion	6.45%
G+	Staphylococcus epidermidis	urine	6.45%
G+	Staphylococcus epidermidis	blood-Anaerobic	3.23%
G+	Staphylococcus aureus	sputum	67.08%
G+	Staphylococcus aureus	Secretion	25.47%
G+	Staphylococcus aureus	Decubitus Ulcers Secretion	3.11%
G+	Staphylococcus aureus	urine	2.48%
G+	Staphylococcus aureus	blood-Aerobic	1.86%
3.2.7 Among the main Gram-negative bacteria, E. coli and Klebsiella pneumoniae are easily detected in sputum and urine. Proteus mirabilis is easily detected in the urine. Pseudomonas aeruginosa and Acinetobacter baumannii were easily detected in sputum (Table 3).

**Table 3:** Distribution of specimens of major Gram-negative bacteria.

Bacterial species	Bacterial name	type of sample	Constituent ratio
G-	E.coli	urine	67.37%
G-	E.coli	sputum	21.75%
G-	E.coli	Secretion	3.86%
G-	E.coli	blood-Aerobic	3.51%
G-	E.coli	blood-Anaerobic	2.46%
G-	E.coli	Decubitus Ulcers Secretion	1.05%
G-	Proteus mirabilis	urine	94.29%
G-	Proteus mirabilis	Secretion	2.86%
G-	Proteus mirabilis	sputum	1.43%
G-	Proteus mirabilis	Decubitus Ulcers Secretion	1.43%
G-	Klebsiella pneumoniae	sputum	59.29%
G-	Klebsiella pneumoniae	urine	33.63%
G-	Klebsiella pneumoniae	Secretion	4.42%
G-	Klebsiella pneumoniae	Decubitus Ulcers Secretion	1.77%
G-	Klebsiella pneumoniae	blood-Aerobic	0.88%
G-	Pseudomonas aeruginosa	sputum	75.45%
G-	Pseudomonas aeruginosa	urine	12.57%
G-	Pseudomonas aeruginosa	Secretion	9.58%
G-	Pseudomonas aeruginosa	Decubitus Ulcers Secretion	2.40%
G-	Acinetobacter baumannii	sputum	77.78%
G-	Acinetobacter baumannii	urine	13.33%
G-	Acinetobacter baumannii	Decubitus Ulcers Secretion	6.67%
G-	Acinetobacter baumannii	blood-Aerobic	2.22%
3.2.8 Among the major fungi, Candida albicans, Candida bulgaricus, and Candida glabrata were easily detected in sputum (Table 4).

**Table 4:** Distribution of major fungi.

Bacterial species	Bacterial name	type of sample	Constituent ratio
Fungus	Candida glabrata	sputum	69.23%
Fungus	Candida glabrata	urine	15.38%
Fungus	Candida glabrata	Secretion	7.69%
Fungus	Candida glabrata	stool	7.69%
Fungus	Candida albicans	sputum	88.89%
Fungus	Candida albicans	urine	5.56%
Fungus	Candida albicans	stool	1.85%
Fungus	Candida albicans	blood-Anaerobic	1.85%
Fungus	Candida albicans	Secretion	1.85%
Fungus	Dubliniensis Candida	sputum	73.33%
Fungus	Dubliniensis Candida	urine	20.00%
Fungus	Dubliniensis Candida	Secretion	6.67%
3.2.9 There was a statistically significant difference in gender distribution and overall gender distribution between Enterococcus faecalis, E. coli, and Pseudomonas aeruginosa in positive samples (Table 5).

**Table 5:** Gender distribution of several bacteria.

Bacterial species	Bacterial name	gender	Frequency	Constituent ratio	χ2
G+	Enterococcus faecalis	female	28	71.79%	4.698∗
G+	Enterococcus faecalis	male	11	28.21%	
G-	E.coli	female	187	65.61%	12.11∗∗
G-	E.coli	male	98	34.39%	
G-	Pseudomonas aeruginosa	male	104	62.28%	16.03∗∗
G-	Pseudomonas aeruginosa	female	63	37.72%	

∗P＜0.05 ∗∗P＜0.01.
3.2.10 There was a statistically significant difference in the distribution of specimens between female specimens and the total specimens (Table 6), χ^2^ = 15.01, P < 0.05.

**Table 6:** Distribution of patient gender and specimen types.

gender	type of sample	Frequency	Constituent ratio	gender	type of sample	Frequency	Constituent ratio	type of sample	Frequency	Constituent ratio
female	urine	276	42.33%	male	urine	156	28.36%	urine	432	35.94%
female	sputum	261	40.03%	male	sputum	323	58.73%	sputum	584	48.59%
female	Secretion	70	10.74%	male	Secretion	30	5.45%	Secretion	100	8.32%
female	blood-Aerobic	20	3.07%	male	blood-Aerobic	18	3.27%	blood-Aerobic	38	3.16%
female	blood-Anaerobic	16	2.45%	male	blood-Anaerobic	9	1.64%	blood-Anaerobic	25	2.08%
female	Decubitus Ulcers Secretion	8	1.23%	male	Decubitus Ulcers Secretion	13	2.36%	Decubitus Ulcers Secretion	21	1.75%
female	stool	1	0.15%	male	stool	1	0.18%	stool	2	0.17%
total		652	100.00%	total		550	100.00%	total	1202	100.00%
3.2.11 There were no statistical differences in the distribution of Gram-positive bacteria, negative bacteria and fungi in each month.
3.2.12 There was a statistically significant difference in the distribution of P. aeruginosa in all age groups, χ2 = 25.70, P < 0.01 (Table 7).

**Table 7:** Age distribution of Pseudomonas aeruginosa.

Bacterial name	Age range	Frequency	Constituent ratio
Pseudomonas aeruginosa	50~59	1	0.60%
Pseudomonas aeruginosa	60~69	26	15.57%
Pseudomonas aeruginosa	70~79	48	28.74%
Pseudomonas aeruginosa	80~89	74	44.31%
Pseudomonas aeruginosa	90~99	18	10.78%
total		167	100.00%
3.2.13 The resistance rate of Staphylococcus aureus to levofloxacin and penicillin was >90%, the resistance rate to clindamycin and erythromycin was >80%, the resistance rate to gentamicin was >70%, and vancomycin and linezolid The amine sensitivity rate was 100.0%, the sensitivity rate to rifampicin and teicoplanin was >90%, and the sensitivity to compound sulfamethoxazole and streptomycin was >80% (Table 8). Staphylococcus aureus resistant to vancomycin and linezolid was not found this time.

**Table 8:** Antimicrobial sensitivity rate and resistance rate of Staphylococcus aureus (%).

Bacterial name type	Staphylococcus aureus	Staphylococcus aureus	Staphylococcus aureus
	I	R	S
Gentamicin	0（0）	116（72.05）	45（27.95）
Compound sulfamethoxazole	0（0）	27（16.77）	134（83.23）
Levofloxacin	0（0）	145（90.06）	16（9.94）
Clindamycin	6（3.73）	132（81.99）	23（14.29）
Rifampin	2（1.24）	7（4.35）	152（94.41）
Ergomycin	2（1.24）	138（85.71）	21（13.04）
Vancomycin	0（0）	0（0）	161（100）
penicillin	0（0）	156（96.89）	5（3.11）
Linezolid	0（0）	0（0）	161（100）
Koalaranin	1（0.62）	0（0）	160（99.38）
Streptomycin	2（1.25）	18（11.25）	140（87.5）

The resistance rate of E. coli to compound sulfamethoxazole was >60%, the resistance rate to cefuroxime, ceftriaxone, levofloxacin, and ciprofloxacin was >70%, and the resistance rate to cefazolin was >80%. The resistance rate to ampicillin is >90%, the sensitivity rate to nitrofurantoin, minocycline and cefoxitin are>60%, and the sensitivity to cefoperazone sulbactam and piperacillin tazobactam is >70%. The sensitivity of ropamine, imipenem, and amikacin was >80% (Table 9).

3.2.14 The resistance rate of Proteus mirabilis to nitrofurantoin was 100%, the resistance rate to ampicillin was >90%, and the resistance rate to compound sulfamethoxazole, levofloxacin and ciprofloxacin was >80%. For cefazolin and gentamicin The rate of resistance is >70%.

**Table 9:** Antimicrobial sensitivity rate and resistance rate of E.coli (%).

Bacterial name type	E.coli	E.coli	E.coli
	I	R	S
Nitrofurantoin	8（4.04）	65（32.83）	125（63.13）
Cefazolin	5（1.77）	238（84.1）	40（14.13）
Gentamicin	0（0）	151（52.98）	134（47.02）
Ampicillin	1（0.35）	262（92.25）	21（7.39）
Cefuroxime	1（0.35）	223（79.08）	58（20.57）
Ceftazidime	13（14.29）	47（51.65）	31（34.07）
Cefepime	38（13.43）	149（52.65）	96（33.92）
Cefoxitin	12（4.35）	95（34.42）	169（61.23）
Ceftriaxone	4（1.41）	209（73.59）	71（25）
Cefoperazone sulbactam	40（14.08）	32（11.27）	212（74.65）
Piperacillin tazobactam	15（5.28）	44（15.49）	225（79.23）
Ampicillin sulbactam	48（16.9）	126（44.37）	110（38.73）
Meropenem	4（1.41）	35（12.32）	245（86.27）
Imipenem	7（2.46）	35（12.32）	242（85.21）
Amikacin	7（2.46）	26（9.15）	251（88.38）
Compound sulfamethoxazole	0（0）	172（60.56）	112（39.44）
Levofloxacin	0（0）	212（74.39）	73（25.61）
Ciprofloxacin	4（1.4）	211（74.04）	70（24.56）
Minocycline	32（11.23）	68（23.86）	185（64.91）

Sensitivity to cefoxitin, imipenem, and amikacin was >80%, and sensitivity to meropenem, cefoperazone sulbactam, and piperacillin tazobactam was >90% (Table 10).

3.2.15 Klebsiella pneumoniae to cefazolin, gentamicin, ampicillin, cefuroxime, ceftazidime, cefoxitin, ceftriaxone, ampicillin sulbactam, compound sulfamethoxazole, levofloxacin, ciprofloxacin The resistance rate was >60%, the sensitivity rate to amikacin was only >60%, and the sensitivity to imipenem and meropenem was only >50% (Table 11).

**Table 11:** Antimicrobial sensitivity rate and resistance rate of Klebsiella pneumoniae (%).

Bacterial name type	Klebsiella pneumoniae	Klebsiella pneumoniae	Klebsiella pneumoniae
	I	R	S
Nitrofurantoin	5（13.51）	21（56.76）	11（29.73）
Cefazolin	2（1.79）	97（86.61）	13（11.61）
Gentamicin	0（0）	74（65.49）	39（34.51）
Ampicillin	0（0）	106（99.07）	1（0.93）
Cefuroxime	0（0）	91（81.98）	20（18.02）
Ceftazidime	1（3.23）	21（67.74）	9（29.03）
Cefepime	12（10.91）	64（58.18）	34（30.91）
Cefoxitin	3（2.78）	71（65.74）	34（31.48）
Ceftriaxone	1（0.91）	83（75.45）	26（23.64）
Cefoperazone sulbactam	8（7.14）	48（42.86）	56（50）
Piperacillin tazobactam	9（8.11）	50（45.05）	52（46.85）
Ampicillin sulbactam	7（6.31）	78（70.27）	26（23.42）
Meropenem	1（0.89）	46（41.07）	65（58.04）
Imipenem	5（4.46）	47（41.96）	60（53.57）
Amikacin	0（0）	38（33.93）	74（66.07）
Compound sulfamethoxazole	0（0）	71（63.96）	40（36.04）
Levofloxacin	5（4.46）	78（69.64）	29（25.89）
Ciprofloxacin	5（4.59）	80（73.39）	24（22.02）
Minocycline	31（28.7）	26（24.07）	51（47.22）
3.2.16 Pseudomonas aeruginosa tested resistance to antimicrobial <50%, sensitivity to ceftazidime, cefepime, meropenem, imipenem, amikacin >60%, sensitivity to polymyxin B >90%, sensitivity to tigecycline was 100% (Table 12).

**Table 12:** Antimicrobial sensitivity rate and resistance rate of Pseudomonas aeruginosa (%).

Bacterial name type	Pseudomonas aeruginosa	Pseudomonas aeruginosa	Pseudomonas aeruginosa
	I	R	S
Gentamicin	3（1.8）	72（43.11）	92（55.09）
Ceftazidime	10（6.02）	51（30.72）	105（63.25）
Cefepime	19（11.38）	37（22.16）	111（66.47）
Cefoperazone sulbactam	34（20.36）	33（19.76）	100（59.88）
Piperacillin tazobactam	28（16.87）	41（24.7）	97（58.43）
Meropenem	8（4.79）	56（33.53）	103（61.68）
Imipenem	3（1.8）	62（37.13）	102（61.08）
Amikacin	10（5.99）	34（20.36）	123（73.65）
Levofloxacin	4（2.4）	92（55.09）	71（42.51）
Ciprofloxacin	6（3.59）	93（55.69）	68（40.72）
Piperacillin	25（14.97）	57（34.13）	85（50.9）
Tobramycin	3（1.8）	65（38.92）	99（59.28）
Aztreonam	36（21.56）	68（40.72）	63（37.72）
Polymyxin B	2（1.2）	4（2.4）	161（96.41）
Tigecycline	0（0）	0（0）	161（100）
3.2.17 The resistance rate of Candida glabrata to fluorocytosine was >60%, the sensitivity rate to amphotericin B, fluconazole and itraconazole was >60%, and the sensitivity to voriconazole was >90% (Table 13).

**Table 13:** Antimicrobial sensitivity rate and resistance rate of Candida glabrata (%).

Bacterial name type	Candida glabrata	Candida glabrata	Candida glabrata
	I	R	S
Fluorocytosine	0（0）	9（69.23）	4（30.77）
Amphotericin B	0（0）	2（15.38）	11（84.62）
Fluconazole	1（7.69）	4（30.77）	8（61.54）
Itraconazole	2（15.38）	2（15.38）	9（69.23）
Voriconazole	0（0）	1（7.69）	12（92.31）

**Table 10:** Antimicrobial sensitivity rate and resistance rate of Proteus mirabilis (%).

Bacterial name type	Proteus mirabilis	Proteus mirabilis	Proteus mirabilis
	I	R	S
Nitrofurantoin	0（0）	64（100）	0（0）
Cefazolin	3（4.29）	53（75.71）	14（20）
Gentamicin	3（4.29）	53（75.71）	14（20）
Ampicillin	0（0）	63（90）	7（10）
Cefuroxime	1（1.45）	47（68.12）	21（30.43）
Ceftazidime	6（23.08）	6（23.08）	14（53.85）
Cefepime	16（22.86）	23（32.86）	31（44.29）
Cefoxitin	3（4.29）	10（14.29）	57（81.43）
Ceftriaxone	1（1.45）	40（57.97）	28（40.58）
Cefoperazone sulbactam	0（0）	2（2.86）	68（97.14）
Piperacillin tazobactam	0（0）	2（2.86）	68（97.14）
Ampicillin sulbactam	9（12.86）	30（42.86）	31（44.29）
Meropenem	0（0）	3（4.35）	66（95.65）
Imipenem	8（11.43）	3（4.29）	59（84.29）
Amikacin	3（4.29）	11（15.71）	56（80）
Compound sulfamethoxazole	0（0）	61（88.41）	8（11.59）
Levofloxacin	1（1.43）	58（82.86）	11（15.71）
Ciprofloxacin	2（2.86）	58（82.86）	10（14.29）
Minocycline	1（1.43）	69（98.57）	0（0）

## 4 Discussion

### 4.1 Reasons for the high proportion of middle-aged and elderly inpatients in nursing hospital

1. Affected by the rapid economic development, increased cost of living, and gradual increase in medical standards, China has gradually entered a stage of low fertility rate, high life expectancy, and high aging [1, 2]. As an economically developed city in China, Shanghai is more aging than other cities in China. The proportion of elderly people over 60 years old is as high as 30.2% [2]. At the same time, the one-child policy is also an influent cause the elderly became a heavy burden for their children [3, 4].

2. The government has set up category such as nursing hosiptal to fit the need of the perspectives of the medical needs of elderly patients and the service contents of general hospitals since 2010.

### 4.2 Reasons for differences between microbial species detected in nursing hospitals and results in Shanghai

According to Yang Yang's '2017 Shanghai bacterial resistance monitoring' study, the total number of Gram-positive bacteria in 49 general hospitals in Shanghai was named Enterococcus, Staphylococcus aureus, and β-hemolytic streptococcus. Gram-negative staphylococci, Streptococcus pneumonia, and Streptococcus mutants. The types and rankings of Gram-positive bacteria detected in this study are partially different from results of Shanghai, and the types of Gram-negative bacteria in this study consistent with results of Shanghai, the rankings are partially different [5]. The patients in the nursing hospital in this study was mostly those over 60 years old, and the patients in general hospitals were evenly distributed in different age groups is the possible causes of differences in the ranking of bacterias. Therefore, the selection of antimicrobial in nursing hospital needs to be considered the actual situation of the local nursing hospital. Because of the limited number of patients in this study, the study of bacterial in nursing hospitals in a large number of patients will be performing in the future.

### 4.3 The significance of gender and microbial species in nursing hospital for clinical intervention

In this study, the proportion of E. coli and Enterococcus faecalis in female patients was twice as high as that in male patients, and the gender distribution of the total study cases was statistically different. It was the most easily detected in urine and the bacteria in the urine of female patients [6, 7], be consistent with the conclusions of Sun Shuhong et al. 2014 ″Analysis of distribution and drug resistance of enterococci in patients with urinary tract infection" [8]. The possible reason is the physiological structure of female patients, long-term hospitalized period, female patients have higher urinary tract infections rate [4, 5, 6, 7, 8]. Clinically, if there are the elderly female who has been bedridden for a long time, accompanied by fever, and diagnosed as urinary tract infections, the antimicrobial which against E. coli and Enterococcus faecalis can be used without the test results in the early stage. Inconsistent with the conclusions of Cao Cuiming and Zhang Xiuping's "Analysis of Clinical Relevant Factors and Drug Resistance of Urinary System Infection Enterococci" [9], the possible reasons is 1. The difference in the different functions of central hospitals and nursing hospital may cause the different age distribution. 2. The sample size of this study is insufficient, and further research will be carried out in the future.

In this study, the proportion of male patients infected with Pseudomonas aeruginosa was nearly twice that of female patients, and the gender distribution of the cases was statistically different. The frequency of Pseudomonas aeruginosa found in sputum was 5 times that of other specimens. And male patients have the highest bacterial positive rate in sputum [10], be consistent with the conclusion of Song Haoyue and Huang Kaifeng, "Clinical Distribution of Pseudomonas Aeruginosa Different Drug-resistant Strains in a Shanghai Hospital from 2014 to 2017" [11]. The possible reasons are cigarette smoking [12], decreased resistance capability in elderly patients [13], and the incidence of COPD is higher than female [14, 15], hypertension [16], the incidence of cerebrovascular accidents is higher than that of female [17], combined with elderly patients with long-term hospitalized period, disturbance of consciousness, using nasogastric tube and other reasons [18]. Clinically, if a male patient is bedridden for a long time, with disturbance of consciousness, accompanied by fever, diagnosed as aspiration pneumonia, pulmonary infection, the antimicrobial which against Pseudomonas aeruginosa can be used without the test results in the early stage.

Besides, the positive rate of bacteria in the urine of female patients is the highest, the rate is 42.33%. urine culture is the primary choice for Female patients, the pros are the procedure of sampling is convenient, no pain and high positive rate [19]. The positive rate of bacteria in sputum was the highest in male patients, and the detection rate was 58.73%. Sputum culture is the primary choice for male patients, which sampling procedure is very easy, no pain and high positive rate [10].

4.4 Pseudomonas aeruginosa has high drug resistance rate, low sensitivity rate, high mortality rate, and hard for curing [20]. It widely exists in natural environment and has strong virulence. It is an important cause for infection, hemorrhoids, abscess and suppurative otitis media [21]. First, in this study, there was a statistically significant difference in the distribution of P. aeruginosa infections among different age groups [22], with 70–89 years old accounting for the highest proportion, account for 73.05%, and 90–99 years old accounting for the least, accounting for 10.78%. according to the Shanghai 2018 Statistical Yearbook, life expectancy at birth is 83.37 years. The possible reasons are bacterial virulence and lung infections in elderly patients [15], and the physical functions are decreasing of elderly patients. Be consistent with the conclusion of Xiao Hongwen's 2017 ″Structure of Pathogenic Bacteria and Risk Factors of Death in Elderly Patients with Severe Pneumonia" [23],elderly patients who have been infected with Pseudomonas aeruginosa have a high mortality rate and need to be treated ASAP. The sensitivity of P. aeruginosa to several major antimicrobial such as ceftazidime, cefepime, meropenem, imipenem, and amikacin are not perform very well in this study [13, 24], If abused, Pseudomonas aeruginosa resistance capability will finally appear [25]. In this study, Pseudomonas aeruginosa was much more sensitive to polymyxin B and tigecycline than the others [26, 27]. At present, only the polymyxin B ointment and tigecycline for injection are available in the Shanghai Medical Insurance Drug List. Due to the limited economic conditions, it is recommended to add the other dosage forms of polymyxin B and lower price of tigecycline.

4.5 Fungal has more chance to detected in sputum. Candida glabrata, Candida albicans, Dubliniensis are common pathogens in fungi, and Candida albicans is more virulence. In this study, all three fungi were detected in sputum, and the possible cause was pulmonary infection caused by fungal pneumonia. The sensitivity of these fungi to fluconazole and itraconazole was acceptable. Amphotericin B and voriconazole are more sensitive [20, 28]. In clinical, sputum is easily contaminated by oropharyngeal colonization bacteria. if the result was fungi in sputum, the test should be reviewed. The sampling personnel should take the training program before taking samples.

4.6 The increased resistance rate of Staphylococcus aureus is one of the most important parts of clinical bacteria control. Staphylococcus aureus is an important pathogen in Gram-positive bacteria, it has high Pathogenicity [29], which is common in skin, nasal cavity, throat, gastrointestinal, sputum, and purulent wounds [30, 31]., be consistent with the conclusion of Li Ling, Lin Zhonghua and others in 2019 ″Analysis of the distribution and drug resistance of nosocomial and community infections of Staphylococcus aureus from 2015 to 2018" [32]. In this study, S. aureus was more common in sputum and secretion. It has produced very strong resistance capability to a variety of antibiotics, which levofloxacin and penicillin are essentially ineffective. The possible cause of the analysis was due to the abuse of β-lactam antimicrobial in the early stage when the drug comes out. At the same time, studies have shown that Staphylococcus aureus has an increasing trend in resistance to compound sulfamethoxazole and rifampicin [33, 34]. the nursing hospital needs to deal with the problems such as long hospital stays, and more patients in the ward and very complex patient's condition. In the future, it is necessary to strengthen the antibiotics training program for doctors and the administrator of the hospital should pay attention to it.

4.7 Intestinal pathogens are common to clinical and food bacterial prevention. E. coli and Proteus mirabilis are conditional pathogens that are widely present in the intestine. The enterotoxin produced by them is an important cause of diarrhea, abdominal pain and fever in elderly patients [35, 36], which can lead to death. In this study, the two strains have detected a strong resistance capability to at are widely present in the intestine. e cause of the analysis is caused by the abuse of β-lactams and cephalosporins. E. coli and Proteus mirabilis are easy to grow in food. In the future, the nursing hospital must strengthen the in-hospital inspection of food hygiene, and strengthen the bacterial prevention capability of cooking, delivering, and distributing [37, 38].

4.8 Klebsiella pneumoniae has a high rate of drug resistance capability, low sensitivity to many antibiotics and it can be transmitted thrugh the respiratory tract, which considers the most difficult part in clinical [39]. Klebsiella pneumoniae is the most important pathogen in the genus Klebsiella. The bacterium has a capsule with strong resistance, rapid growth and reproduction, and lower resistance capability of elderly patients [40], Conclusions consistent with the 2017 ″Clinical Distribution 2013–2015 1830 Klebsiella pneumoniae infection and drug resistance," Zhanling Ling, Huang Haixia, etc [41]. The possible reasons are the bacteria can be transmitted through the respiratory tract, and the nursing hospital patients have a long hospital stay and a high exposure risk. Once the infection is happening, the progress running rapid and the mortality rate is very high. In this study, Klebsiella pneumoniae has shown the resistance to all tested antimicrobial which is cefepime, cefoperazone sulbactam, piperacillin tazobactam, meropenem, imipenem. there are not any choice available for doctors in the nursing hospital. It is the most important part that the administration should pay attention to [15].

### 4.9 Interventions

Since the daily care of nursing hospitals are mainly carried out by the care workers, which are an important part of personnel in the nursing hospitals. The people do not have the medical background, sometimes they are exaggerating the harm of bacteria. It is very easy to create a panic in the nursing hospital. Therefore, at present, the following interventions are carried out in a nursing hospital on a small scale.

#### 4.9.1 Intervention

4.9.1.1 Adjust the hospital antimicrobial drug list. add drugs into the list: teicoplanin for injection, enteric-coated tablets for nitrofurantoin, piperacillin sodium tazobactam for injection, tigecycline for injection, amphotericin B liposomes for injection, Itraconazole dispersible tablets, the antibacterials which are essentially ineffective remove from the list: clindamycin phosphate for injection, cefuroxime sodium for injection.
4.9.1.2 the result of the bacteriological investigation is conducted and published monthly.
4.9.1.3 Isolating the patient and perform the air sterilizer protocol daily in the specific ward.
4.9.1.4 Strengthen the protection for the medical personnel and increase the implementation of hand hygiene procedure.
4.9.1.5 Strengthen training on infection prevention and control knowledge. The training of this knowledge for care workers is easily ignored. While strengthening the training for the care workers is a very effective way to prevent cross-infection in the ward.
4.9.1.6 Increase air ventilation.
4.9.1.7 Inform the specific patient's information to the medical personnel ASAP.

#### 4.9.2 Intervention results

After 3 months of intervention, some goals were achieved. Firstly, the illness can be treated in the early stage. Secondly, the cost can be reduced. Finally, increase the prevention awareness among medical personnel in a nursing hospital.

### 4.10 Reasons for invalid training results

In clinical, the results of ineffective culture are more common in the following reasons: overdue the time period of sampling, skin is contaminated during sampling, samples are taken after using antimicrobial drugs, body temperature does not meet the requirements, the oral cavity is not cleaned, sampling from the lower part of the urine collection bag, sampling from the purulent secretion on the surface of the hemorrhoid ulcer, lack of sampling techniques, Lack of responsibility.

## 5 Outlook

At present, compared with general hospitals, the nursing hospital is faced with complicated patients and very few antimicrobial drugs, lack of policy, lack of funds, lack of medical personnel, and the patients family members lack the willingness to pay the bill for the treatment. it is expected that the departments will give greater policy and funding support to the nursing hospital's prevention work in the future. At the same time, the nursing hospital should also increase prevention capability, improve the training program. Also expected that the society can strengthen prevention propaganda and moral education, improve the willingness of patients' families, and give more understanding and support for the nursing hospitals.

## Declarations

### Author contribution statement

Y. Wu: Conceived and designed the experiments; Performed the experiments; Analyzed and interpreted the data; Wrote the paper.

J. Xu: Conceived and designed the experiments; Contributed reagents, materials, analysis tools or data; Wrote the paper.

### Funding statement

This research did not receive any specific grant from funding agencies in the public, commercial, or not-for-profit sectors.

### Competing interest statement

The authors declare no conflict of interest.

### Additional information

No additional information is available for this paper.

## References

[1] Li Ji, Che Tingting (2019). International empirical study on the relationship between aging, declining birthrate and savings rate. Shanghai Finance.

[2] Shi Yufeng, Du Xiaolei (2016). Analysis of the supply and demand characteristics of the long-term nursing market in Shanghai. Smart Health.

[3] Zhou Liqun, Zhou Xiaobo (2016). An institutional economics analysis of the decline in fertility in China——an explanation from the socialization of old-age care. J. Southwest Univ. Natl. (Humanit. Soc. Sci. Ed.).

[4] Yu Wei, Liu Baihui (2012). Construction of China's elderly care service system and forecast of demand—taking Shanghai as an example. J. Popul. Sci..

[5] Yang Yang, Guo Yan, Zhu Demei (2019). etc. Surveillance of bacterial resistance in Shanghai in 2017. Chin. J. Infect. Chemother..

[6] Tao Xiaoyan, Tang Rong, Yi Junwen (2018). Drug resistance monitoring of clinically isolated bacteria in urine samples. Chin. J. Infect. Chemother..

[7] Xing Huang, Dong Liang, Zhang Xiuhong (2018). Distribution of pathogens and drug resistance analysis of 10042 urine culture specimens of elderly women in a hospital in Wuxi City. Mod. Prev. Med..

[8] Sun Shuhong, Hu Xiaofeng, Feng Shangcai (2014). Distribution Characteristics and Drug Resistance Analysis of Enterococci in Patients with Urinary Tract Infection.

[9] Cao Cuiming, Zhang Xiuping (2018). Analysis of clinical related factors and drug resistance of urinary system enterococci. J. Community Med..

[10] Chen Yongjun, Chen Yongmei, Zhang Liqun (2018). Distribution and drug resistance analysis of Pseudomonas aeruginosa in a teaching hospital in Shenyang, 2015-2017. China Med. Innovation.

[11] Song Haoyue, Huang Kaifeng, Tang Rong (2019). Clinical Distribution of Different Drug-Resistant Strains of Pseudomonas aeruginosa in a Hospital in Shanghai in 2014-2017.

[12] Lin Lina, Chen Liping (2019). Effects of smoking on IL-4 and IL-17 in induced sputum of patients with lower respiratory tract infection. Chin. J. Microecol..

[13] Yu Guangchao, Li Juxiang, Liu Juzhen (2011). Drug resistance status and outer membrane pore protein analysis of Pseudomonas aeruginosa. J. Jinan Univ. (Natural Sci. Med. Ed.).

[14] Liu Meishan, Huang Kewu (2019). Infection and acute exacerbation of chronic obstructive pulmonary disease. J. Clin. Pulm. Med..

[15] Yonggang Wei, Yan Hongying, Xiao Yuanhong (2018). Analysis of pathogenic bacteria distribution and high-risk factors of acute exacerbation of chronic obstructive pulmonary disease and pulmonary infection in the elderly. Chinese J.Mycol..

[16] Yang Xuetao, Zhou Liang, Liu Fangzhou (2019). Gender difference and mechanism of hypertension incidence. Chinese J. Hypertens..

[17] Wen Yumei, Liu Xiujuan, Liu Xiaoyu (2016). Correlation between aortic arch atherosclerosis and stroke. J. Hebei North Univ. (Nat. Sci. Ed.).

[18] Peng Jinghong, Hou Yanqiang, Zheng Rong (2015). Drug resistance analysis of Pseudomonas aeruginosa. Chinese J. Hosp. Infect..

[19] Xu Ruiling (2016). Analysis of 52 cases of type 2 diabetes with urinary tract infection in the elderly. J. Xiangyang Vocational Tech. College.

[20] Xu An (2015). Retrospective Analysis of Voriconazole in the Treatment of Invasive Fungal Infections in Elderly Patients.

[21] Wen Liu, Wu Zhi (2013). Clinical distribution and drug resistance analysis of pathogens in sputum culture specimens in our hospital. J. Microbiol..

[22] Liang Bishan, Qi Chen, Li Libing (2019). Pseudomonas aeruginosa infection and drug resistance analysis in elderly inpatients. Int. J. Lab. Med..

[23] Xiao Hongwen (2017). Composition of pathogenic bacteria and risk factors of death in elderly patients with severe pneumonia. Chinese J. Gerontol..

[24] Yuan Li, Xu Yuzhong, Cheng Minggang (2018). Carbopenem-resistant Pseudomonas aeruginosa resistance status and integron resistance gene analysis. China Medical Equipment.

[25] Dong Mengmeng, Liang Qingqing, Zhang Huiqun (2016). Novel β-lactamase-mediated carbenicillin resistance in Pseudomonas aeruginosa. J. NW Univ..

[26] Yuan Ying, Yang Ying, Polymyxin B. (2017). How to choose. Chin. J. Infect. Control.

[27] Zheng Qiaowei, Luo Sasai, Ren Xiaodong (2018). Analysis of related factors of tigecycline exposure to Pseudomonas aeruginosa infection. Chin. J. Hosp. Pharm..

[28] Fan Hongshun, Xiong Zhijiao, Lin Peng (2019). Clinical effect and imaging analysis of fluconazole and voriconazole in the treatment of severe liver disease complicated with pulmonary fungal infection. Chinese J. Hosp. Infect..

[29] Xie Yanbin (2018). Distribution characteristics and drug resistance analysis of Staphylococcus aureus from different sources. Laboratory Med. Clin. Med..

[30] Liu Xiaoqi, Yu Hua, Huang Xiangning (2012). Clinical distribution and drug resistance analysis of Staphylococcus aureus. Sichuan Med. Univ..

[31] Fu Baoqing, Qi Wenjing, Hu Chunling (2017). etc. Drug resistance analysis of Staphylococcus aureus from 2013 to 2015 in Daqing area. Chin. J. Sci. Instrum..

[32] Ling Li, Lin Zhonghua, Lu Weihong (2019). Distribution and drug resistance analysis of nosocomial and community infections of Staphylococcus aureus from 2015 to 2018. Chinese J. Disinfect..

[33] Hu Zhijun, Pan Kai, Pan Xiaolong (2019). Surveillance of bacterial resistance in a top three hospital in Anhui Province in 2017. Chin. J. Antibiot..

[34] Zhou Wenjing (2012). RpoB Gene Mutation and Molecular Epidemiology of Rifampin-Resistant Staphylococcus aureus.

[35] Zhang Ping, Yang Haoliang, Bojun Zhen (2019). Surveillance Analysis of Bacterial Pathogen Spectrum of Infectious Diarrhea in Tongzhou District, Beijing, 2016-2018.

[36] Shi Xiaolu (2016). Research and Application of Virulence Factors of Proteus mirabilis Causing Diarrhea.

[37] Yao Lili, He Ping, Shen Xianbiao (2017). Analysis of monitoring results of microbial contamination in foods in Baoshan District, Shanghai, 2014-2016. Mod. Prev. Med..

[38] Huo Zhe, Bai Shuyuan, Gao Bo (2014). Analysis of food poisoning incidents of Vibrio parahaemolyticus and Proteus mirabilis detected together. Chinese J. Hygiene Inspection.

[39] Wang Ruihua, Feng Heqiang, Zhao Yiming (2019). Analysis of the etiology and risk factors of bloodstream infection in elderly patients. Chin. J. Antibiot..

[40] Du Fangling, Mei Yanfang, Wan Lagen (2018). Serotyping of capsular serotype of Klebsiella pneumoniae and carbapenem resistance in high mucus type. Chin. J. Infect. Chemother..

[41] Zhan Lingling, Huang Haixia, Chen Yuehui (2017). Clinical distribution and drug resistance analysis of 1830 Klebsiella pneumoniae infections from 2013 to 2015. Chinese J. Clinical Pharmacol..

